# Psychiatric Patients Experiences with Mechanical Restraints: An Interview Study

**DOI:** 10.1155/2015/748392

**Published:** 2015-06-25

**Authors:** Klas Lanthén, Mikael Rask, Charlotta Sunnqvist

**Affiliations:** ^1^Skåne University Hospital, Södra Förstadsgatan 101, 214 28 Malmö, Sweden; ^2^Department of Health and Caring Sciences, Linnaeus University, Lückligs Plats, 351 95 Växjö, Sweden; ^3^Department of Care Science, Faculty for Health and Society, Malmö University, Jan Waldenströms Gata 25, 205 06 Malmö, Sweden

## Abstract

*Objective*. To examine psychiatric patients' experience of mechanical restraints and to describe the care the patients received. *Background*. All around the world, threats and violence perpetrated by patients in psychiatric emergency inpatient units are quite common and are a prevalent factor concerning the application of mechanical restraints, although psychiatric patients' experiences of mechanical restraints are still moderately unknown. *Method*. A qualitative design with an inductive approach were used, based on interviews with patients who once been in restraints. *Results*. This study resulted in an overbridging theme: *Physical Presence, Instruction and Composed Behaviour Can Reduce Discontent and Trauma*, including five categories. These findings implicated the following: information must be given in a calm and sensitive way, staff must be physically present during the whole procedure, and debriefing after the incident must be conducted. *Conclusions*. When mechanical restraints were unavoidable, the presence of committed staff during mechanical restraint was important, demonstrating the significance of training acute psychiatric nurses correctly so that their presence is meaningful. Nurses in acute psychiatric settings should be required to be genuinely committed, aware of their actions, and fully present in coercive situations where patients are vulnerable.

## 1. Introduction

All around the world, threats and violence (termed aggressive incidents) perpetrated by patients in psychiatric emergency inpatient units are quite common and are prevalent factors concerning the application of mechanical restraints [1–3]. Aggressive incidents are attributed to numerous causes, such as frustration, pathology, or staff influence on the environment or even staff, patient relationships [4–7]. The majority of the patients subjected to mechanical restraints in Sweden are diagnosed with borderline personality disorder and suffer from self-injury behaviour patterns [8]. In Norway, two separate studies conducted in psychiatric inpatient units from different hospitals found that mechanical restraints were most commonly used on young, male patients [9, 10].

A Polish study found psychotic episodes to be the most common diagnosis for patients in mechanical restraints [11]. In addition, a Japanese study [12] found that restrained patients are significantly more likely than nonrestrained patients to be diagnosed with organic mental disorders or substance disorder and schizophrenia but are less likely to be diagnosed with a mood disorder or a neurotic disorder.

In 2011, mechanical restraints were used 3,400 times on 1,142 patients in Swedish psychiatric inpatient units in connection to violence or self-harm. In 2012, mechanical restraints were used 4,123 times on 1,327 individuals and hence 2156 times on women and 1967 times on men. The statistics showed that it was common that one patient was placed in mechanical restraints several times and it was more common among women. The use of mechanical restraints seems to have increased in Sweden between 2010 and 2013 [8].

In Scandinavian countries, systematic risk assessments to predict aggressive behaviour or special methods for handling aggressive patients have been implemented in several psychiatric inpatient wards [6, 13]. Assessment aims are to avoid outbursts of aggression, to minimize risk of injury, to reduce the need for restrictive coercive measures, and to help the patients control their behaviour.

Although coercive measures occur [14], research into patients' experiences is rare. There are some studies describing both negative and more neutral reactions. Chien et al. [15] found from interviews that patients felt afraid, humiliated, or being punished having been placed in mechanical restraints. Some patients felt more frustrated and found that mechanical interventions brought back frightening memories; many studies have stated that mechanical restraints elicit traumatic memories [16–21]. Less negative or neutral reactions sometimes occurred when the health professionals were able to provide psychological and informational support to patients throughout the mechanical restraints [15].

Nurses in psychiatric intensive care units are frequently confronted with patient aggression, and many nurses in such settings have experienced patient violence during the course of their careers [22]. Nurses in general do not experience the use of coercion as positive [23], but at times they still need to perform coercive measures. According to Olofsson et al. [23], nurses understand the necessity of performing coercive measures, but at the same time they ought to perform the coercive measure as a nonoffensive action. A Norwegian study about staff attitudes to coercion in psychiatric inpatient units [24] showed that male and unskilled staff were significantly more prone to use a highly restrictive intervention against violent patients than the rest of the staff.

Both patients and staff describe their relationship as an important issue [5, 25]. The interpersonal relationship and being a human are two important issues between the nurse and the patient when a coercive measure is performed [25]. Carlsson [5] found that this was explicated by seven themes of meaning: respecting the individual's fear and respecting the client, touch, dialogue, situated knowledge, stability, mutual regard, and pliability.

Quality and Safety Education for Nurses (QSEN) has described six core competencies for advanced nursing [26], and the overall goal is to prepare future nurses to continuously improve the quality and safety of the healthcare system. Person-centred care, one of the six core competencies, aims to provide compassionate and coordinated care based on respect for patients' preferences, values, and needs. The therapeutic relationship between the psychiatric nurse and the patient is at risk when applying mechanical restraints [27]. Consequently, it is also important to examine this interaction further. Many studies on coercive measures focus on the nurses' experience and official reports of violent incidents [28]; thus, psychiatric patients' experiences of mechanical restraints are still moderately unknown. Their experiences are the main factors that may improve care when coercive measure ought to be performed. Therefore, the aims of this study are to examine psychiatric patients' experience of mechanical restraints and to describe the care the patients received.

## 2. Methods

### 2.1. Design

The restrained individuals themselves can only answer what it actually means to be fixed in restraints. Consequently, a qualitative design with an inductive approach based on interviews was chosen.

### 2.2. Participants and Data Collection

Psychiatric outpatient units, as well as patient organizations, were contacted by mail or visited by the first author. Posters giving information about the study were placed at outpatient units and at the houses of patient organization. Patients from inpatient units were not included. The reason for this was to avoid informants with current acute mental illness. Inclusion criteria included proficiency in the Swedish language and having been mechanically restrained at least once, while the exclusion criteria included those less than 18 years of age and those who currently had been admitted to a psychiatric inpatient unit because of acute mental illness.

The participants responded in different ways: two participants responded by e-mail, two responded by telephone, two participants gave verbal consent through their contact person at the outpatient unit so that the first author could contact them, and two participants gave verbal consent at a patient organization to the first author. Three participants were contacted by the first author because two participants gave verbal consent to the first author through another informant, while one gave verbal consent through the third author. All participants provided written, informed consent before the interview, having received verbal information from the first author.

The participants constituted ten former psychiatric patients (five men and five women) who were currently, or had been previously (at some point in life), subjected to compulsory psychiatric care and who had experienced mechanical restraints. The ages ranged from 32 to 70, and the mean age was 47.3 ± 14.3 years. By their own admission, the participants had been treated for psychosis, self-harming, and bipolar disorders.

The informants themselves decided the time and location of the interviews. The one-on-one interviews began with demographic questions followed by two open-ended questions, such as* “Can you describe for me how the restraints procedure was performed and how you experienced the situation?” *followed by* “How did the staff act and how did you want them to act?”* The interviewees were encouraged to talk freely to enrich their story. However, the author also had an interview guide with supportive questions to use if the informants found it difficult to speak of their own accord. The interviews were conducted during Spring Term 2013 and were duly recorded. The median time for an interview was 24 minutes, ranging from 12 to 45 minutes, and was transcribed verbatim by the first author. One (of eleven interviews) was excluded due to a tape recording failure.

### 2.3. Analysis

The interviews were analysed using quantitative content analysis according to Miles and Huberman [29] and Berg [30]. The use of both manifest and latent analyses facilitated the apparent structure, as well as the deep structural meaning of the interviews. Both the first and the third authors read the transcribed material separately. The first author read the transcriptions several times and identified meanings units, which described how psychiatric patients' experienced the mechanical restraints. After the meanings units were identified, they were condensed into codes. The process continued with the organization of the subcategories into categories. Both the first and the third authors separately formulated subcategories into categories, and consensus was extended after comparison and discussions. Five categories were identified after comparisons, and finally a theme was formulated.

The first author has experienced being a nurse at an intensive psychiatric care unit and had acted as a team leader in many situations where mechanical restraints had been used. The author brought his preconception of the experience of patients who have been in restraints into the interviews. All authors have worked for several years as psychiatric and mental health nurses, and they also have deeper knowledge about the context.

### 2.4. Ethical Considerations

Ethical approval was granted from the Ethical Advisory Board at Malmö University, Department of Health and Society, Malmö, Sweden (Dnr: HS60-2012/1047:13). Participants were told that they could opt to discontinue the study at any time before, during, or after the interviews. All participants gave both verbal and written informed consents.

## 3. Results

The findings are presented within the structure of a category system based on an overall theme entitled* Physical Presence, Instruction and Composed Behaviour Can Reduce Discontent and Trauma*, which is based on the categories created from the subcategories (Table 1).

### 3.1. Physical Presence, Instruction and Composed Behaviour Can Reduce Discontent and Trauma

The importance of an appropriate attitude and care from staff when placing the patient in mechanical restraints was stressed repeatedly in the interviews; in many cases, it was the determining factor as to how the mechanical restraint situation was experienced. Different dimensions of attitude and actions arose from the narratives. The informants pointed out the significance of receiving clear information during the mechanical restraints situation. Having knowledge about what was happening and what was going to happen gave the participants a sense of control, calm, and security. It was suggested that this information should be given by one person only during the mechanical restraints situation. Clear information was described by some informants as being the most important aspect of quality care in a mechanical restraints situation. The interviews also revealed that the physical presence of staff was crucial for the patient's positive experience during his or her time in mechanical restraints. Feelings of safety and warmth in connection to the presence of a staff were reported.

If the nursing care during the mechanical restraints situation was carried out according to the above-mentioned criterion, there was a chance for a positive experience from the procedure for the patient. The mechanical restraints situation would, in such cases, perhaps not lead to discontent or traumatisation.

### 3.2. Safety and Understanding

Several informants reported feelings such as calm and safety from the mechanical restraints situation. Being restrained was described as security (safety) because it prevented the patient from self-harming behaviour. It was also described as an ultimate and resolute method of stopping a growing feeling of loss of control and degeneration. The calmness emerged from the feeling of safety. The informants perceived the calmness as a positive feeling in a situation that was otherwise unusual and unpleasant:

*That part I remember quite a lot of was not who did it and so on, but just that they did it and that it made me feel safe. (Interview 3)*

Thoughts and feelings about the mechanical restraints as necessary and inevitable reappear. The mechanical restraints were sometimes described as unavoidable or essential. The informants had, in these cases, an understanding of the situation and that it perhaps was the only option left:

*It was, it was like the only way there was, so to speak, since nothing else worked. I wasn't able to speak, so it was a little hard to, like, just take a few deep breaths. (Interview 1)*

Mechanical restraints were defined as an intervention that becomes necessary for calming a person who is extremely anxious and violent. Therefore, some informants regarded it as an intervention that fulfilled a purpose.

### 3.3. Fear, Powerlessness, and Feelings of Unreality

The fear that the mechanical restraints sometimes reappears in many of the interviews. In some descriptions, the fear was manifested as anger and as a feeling of violation. One of the informants described a feeling of pure fear in connection with the mechanical restraints. The fear was described as a strong negative feeling and was many times characterised as one of the worst fears they had ever experienced:

*It's mostly fear really, a real horror show, it was terrible. (Interview 7)*

*I have never been so afraid in my entire life. (Interview 4)*

The fear was often caused by a sense of ambiguity about what was going to happen next and how long the mechanical restraints would stay in place. The patients often lost the concept of time in the mechanical restraints room, and this led to anxiety.

Being in mechanical restraints also led to a feeling of powerlessness and being excluded or exposed. The feeling was partially connected to the feeling of insecurity, but it was also manifested as an experience of its own. Some informants described a total loss of control and how they experienced a position of dependence on the staff. The feeling was experienced as negative, and the idea of no longer having control over what was going to happen was perceived as frightening:

*To be left to someone else's good will because you do not have, sort of, it's not possible for you to get up, you cannot talk yourself out of the situation, you cannot sort of…. (Interview 1)*

Feelings of unreality occurred for some informants when they were in mechanical restraints. They described what was going on as absurd and different, so much that they could not really take it in:

*It felt a bit like a movie, sort of, eeeh it felt a bit like, sort of eeeh, it's just like this sort of, this is just fake almost kind of… this is just not real. (Interview 2)*

The difficulties in taking in what was happening were sometimes described as a sudden flight and a screen from reality. The feelings were described as a condition of splitting and sudden disintegration.

### 3.4. Composed and Professional Attitude

It appears that the informants experienced the staff in different ways during the mechanical restraints procedure. Both negative and more beneficial experiences of the staff's attitude and care were noted.

The informants described a composed, soft, and well-balanced attitude and care as well as actions, which often resulted in a dignified and respectful “care” during the mechanical restraints procedure. The care was described as nonhectic, and it helped the person to calm down or to stabilize an already escalated and troublesome situation:

*When, with a shit load of people, fix what they are supposed to fix and then out again fast and then there were only the few left to carry on further contact with me, so it was very, it was like an isolation measure, sort of. Everyone in and step on the gas and then out again. So it became as calm as possible as fast as possible, so I think that it was quite professionally done actually. (Interview 1)*

Experience of an emotionally cold and clinical attitude, as well as behaviour, also emerged from the interviews. Impersonal and negligent behaviour concerning the feelings of the person in restraints was described: a procedure where barely a word was uttered and the staff acted out of routine. If something was said, it was said in a negative and accusatory tone. One informant even experienced that she had been taunted and verbally ridiculed by the staff under restraint. Many times, it emerged from the informants that staff had been talking* about *them rather than* with* them or* to* them:

*Everything happens in total silence and eh it's sort of just, the only thing that happens is that you feel that they are talking over your head; you just hear, “Yes you take that one there and you take this and have you got that buckle?” And stuff like that. (Interview 9)*

In several interviews, the informants corroborated that the attitude and level of care of the staff were significant to how they experienced the situation. If the staff kept calm, acted in a stable manner, and managed to deal with their feelings, the mechanical restraints situation was experienced as quite neutral and, in retrospect, meaningful. It could, in some cases, have also been avoided. If the staff, on the other hand, acted unprofessionally, with a cold and harsh attitude, this affected the experience of the mechanical restraints situation negatively. Furthermore, this could affect the entire experience of the hospitalization period and lead to increased suspiciousness towards psychiatry in general:

*I remember it was a total horror show, and then you get imprinted by that if you think about the psychiatry and so how you perceive the psychiatry and doctors and all that; you are a little cautious with doctors what you tell them and such, so they do not misinterpret you. (Interview 7)*

In addition, many informants described the mechanical restraints situation as traumatic. They believed that they had been so frightened and violated by the incident that it had marked them. Some described sleeping problems and that they thought about the mechanical restrain situation on a daily basis. They could feel poorly long after the incident when they thought about it, and it had created worry and feelings of fear within them. Thoughts about disproportionate measures in psychiatry emerged and the restraining procedure had not been performed the way it should have been according to directions:

*This is probably, I believe, quite a major intervention and too many actually. Maybe then especially if you are, so to say, “clear in the head” when it happens and so and that you remember it, then it becomes kind of like a trauma. (Interview 4)*

A point of view that permeated almost all the interviews was that the negative reactions to the mechanical restraints situation could have been prevented. It could have been avoided with the use of a professional, composed, and stable attitude and quality care from the staff.

### 3.5. Physical Presence and Giving Information

The importance of physical presence and contact just after the mechanical restraint was performed and emphasized in several interviews. The presence of the staff during the time in restraint was important for informants' feeling of safety. If the staff sat at a distance from the restrained person and did not engage sufficiently with the patient, this was experienced as negative. Contact with staff and being active were described as important. Physical contact, such as placing a hand on the shoulder or on the arm, could additionally contribute to the feeling of safety, decrease anxiety, and induce calm:

*A psychiatric nurse was left, and he touched my arm and just asked if there was something he could do for me; and then I said yes you can keep holding your arm there, because it felt so safe. (Interview 2)*

The experience of vagueness in connection with the mechanical restraint has shown itself to be troublesome for many of the informants. In many interviews, the wish to be informed about what was going to happen next emerged. Clear information from the staff about their intentions in connection with the mechanical restraint was requested. Information and communication with, preferably, one person during the procedure were the best according to many informants. Knowing what was going on, what the staff were doing with them, and how long they were going to be restrained provided a sense of safety. In the interviews, the fact that the informants had not always been told what was going to happen and that the staff had not communicated with them emerged. Many of the informants stated that it was important to get information during the procedure about how and why the mechanical restraint was being performed:

*I think that it is enormously important that you get to know what is going on, that you, that someone tells you what is going on and what you are supposed to do. (Interview 8)*

### 3.6. Debriefing and Processing

The informants expressed a desire to talk about their experience in the mechanical restraints situation. The possibility of debriefing with staff was seen as a way to process the experience. If an understanding of the situation that occurred could be reached through hearing the staff's version, the processing of the experience was supported. A successful adoption of the situation was crucial if the experience was to develop into trauma or not. The informants proposed debriefing as a good method. It included both a verbal follow-up and a written follow-up with the staff, with the written follow-up being in the form of a questionnaire. Several informants saw a later reduction of the drama of the mechanical restraints situation as essential, so that they could move on from the experience:

*After experiencing a mechanical restraints situation like this, I think it's really good to have a debriefing session just like after an incident. (Interview 4)*

## 4. Discussion

This study resulted in an overall theme:* Physical Presence, Instruction and Composed Behaviour Can Reduce Discontent and Trauma*. The results disclosed that the physical presence of staff was crucial for the patient's positive experience during his or her period in mechanical restraints. An appropriate attitude and level of care from the staff when placing the patient in mechanical restraints were the determining factors concerning how the situation was experienced. The informants pointed out the significance of receiving clear instruction during the mechanical restraints situation. Receiving knowledge about what was happening and what was going to happen provided the participants with a sense of control, calm, and safety. Similar results have been described previously; for example, [15, 31, 32] have shown how a calm and professional attitude towards patients in a mechanical restraints situation increases the chances of a positive experience for the patient. The importance of giving clear information to the patient has also been reported [15, 33]. The value of the presence of a staff member has partially been reported [32]. The essential issue in the present study was the importance of the physical presence of a supporting staff during the procedure and the debriefing procedure afterwards. Otherwise, as this study has shown, the mechanical restraints can be experienced as abusive and, in some cases, as creating trauma.

Consequently, it is important to identify risk patients so that coercive restraint can be prevented. In Sweden, a noncommercial violence prevention and management programme has been introduced. This model was originally developed in Haukeland University Hospital, Department of Forensic Psychiatry, Bergen, Norway [13]. Among other things, this model emphasises that the staff maintain neutral body language and a calm tone when communicating with a patient exhibiting aggressive behaviour. This approach demonstrates that the staff are not seeking confrontation with the patient. Similar guidelines are used in the Project BETA (Best practices in Evaluation and Treatment of Agitation), a noncoercive deescalation with the goal to calm an agitated patient and to gain his or her cooperation in the evaluation and treatment [34]. There is strong international focus on reducing and possibly eliminating restraint [35–37] which is important and necessary, but still mechanical restraints occur, and some are unavoidable considering the violence-controlling methods that exist today.

Several participants in the study explained that they thought that mechanical restraint was necessary in certain extreme situations, and they expressed an understanding for the use of this intervention. The patients described a perceived feeling of safety because staff were helping them to control their aggressive symptoms, that is, to be put in restraints to prevent behaviour dangerous to other patients or to staff [15, 38]. A competency clarified by QSEN [26] is safety, which means minimising risk of harm to patients and care providers through both system effectiveness and individual performance. Therefore, there must be skills present to establish effective use of strategies in order to maintain safety and understanding when mechanical restraint is necessary, for example, to communicate and inform the patient throughout the entire procedure with a calm voice, as well as being physically present.

The interviews in this study describe varying quality in staff attitude and care towards patients during the mechanical restraints procedure. Informants described both gentle and not-so-gentle approaches. If the staff kept their calm, acted in a stable manner, and managed to deal with emotions, the mechanical restraints situation felt satisfactory for the patients and, in retrospect, meaningful. If the staff, on the other hand, acted unprofessionally, being cold and harsh in their attitudes and care, this affected the experience of this situation negatively.

The diversity of the care described during mechanical restraint shows the importance of further discussions on the standardization of safety for patients when using mechanical restraints. Here, it also becomes important to apply the QSEN [26] competency: person-centred care in order to provide compassionate and coordinated care based on respect for patients' preferences, values, and needs.

In order to reduce negative feelings, it was important that staff were present during the mechanical restraints period. The physical presence of staff was described as significant and was seen as important for feelings of safety. To the best of the authors' knowledge, this has been only ambiguously reported previously. The expressed need of receiving information before and during the mechanical restraints procedure and the significance of the staff communicating with patients was another important component in this study. Another important factor was to be able to discuss the process and the experience of mechanical restraints with the staff after the event. The opportunity of debriefing with staff was seen as a way to adapt to the experience. This included both a verbal follow-up and a written follow-up with the staff. Similar findings are reported by [21, 39], who came to the conclusion that debriefing after mechanical restraints was essential for the patient and also for the staff, so they could jointly ascertain how mechanical restraints could be avoided in the future.

This study is important as it confirms earlier studies' findings of negative feelings, importance of communication, and debriefing in connection with mechanical restraints [13, 15, 16, 19, 22, 32, 39–41]. It also brings new knowledge with the narrated importance of physical presence of staff when mechanical restraints are deemed inevitable.

## 5. Limitations

There are several limitations to this study, for example, the small sample of ten informants and the fact that all informants came from outpatients units. Consequently, the informants gave their narratives retrospectively and might have reconstructed their experiences. It might have been different if the narratives came from inpatients with a fresh memory of the event. The difficulty with such a method was to obtain ethical permission for research on patients in compulsory institutional care. Five of the ten interviews were coded by the first and third authors, leaving five interviews coded solely by one researcher. Yet, we aimed to achieve reliability in agreement with the standard criteria for qualitative research [42].

## 6. Conclusions

When mechanical restraints are unavoidable, a nursing perspective must be taken into consideration. Information must be given in a calm and sensitive manner, the staff must be physically present during the entire procedure, and debriefing must be conducted thereafter. If this is accomplished, the overall goals of QSEN to improve the quality and safety of healthcare systems are on their way to be achieved [26]. The importance of the presence of committed staff during mechanical restraints that this study shows demonstrates the significance of training acute psychiatric nurses correctly so that their presence is meaningful. Nurses in acute psychiatric settings should be required to be genuinely committed, be aware of their actions, and be fully present in coercive situations where patients are vulnerable. This entails that managers of acute psychiatric settings ensure that staff get appropriate training in empathy and violence prevention. Furthermore, this could form the basis of consensus when creating nationwide routines for nursing care in mechanical restraints situations.

## Figures and Tables

**Table 1:** Theme and categories.

Theme	Categories	Subcategories
Physical Presence, Instruction and Composed Behaviour Can Reduce Discontent and Trauma	Safety and understanding	Positive emotions Feeling of necessity

	Fear, powerlessness, and feelings of unreality	Negative emotions Uncertainty Feelings of unreality and splitting

	Composed and professional attitude	Impersonal and clinical approach The attitude is important Risk for trauma Calm and sober behaviour

	Physical presence and giving information	Communication is important Presence and body contact

	Debriefing and processing	Follow-up interventions
